# The Impact of the COVID-19 Pandemic on the Functionality of International Surgical Volunteer Organizations

**DOI:** 10.3389/fsurg.2022.868023

**Published:** 2022-04-06

**Authors:** Spencer Lyons, Amy L. Xu, Wesley M. Durand, Shyam Patel, Julius K. Oni, Jacob M. Babu

**Affiliations:** ^1^The Warren Alpert Medical School of Brown University, Providence, RI, United States; ^2^Department of Orthopaedics, Johns Hopkins University, Baltimore, MD, United States

**Keywords:** COVID-19, surgical volunteer organizations, international healthcare, low- and middle-income countries (LMIC), survey

## Abstract

**Background:**

Surgical volunteer organizations have been severely limited during the ongoing coronavirus disease pandemic. Our purpose was to identify obstacles to surgical volunteer organizations secondary to COVID-19 and their responses.

**Methods:**

Forty-one surgical volunteer organizations participated in a web-based survey (156 invited, 26% response rate). Respondents were separated into two groups: low donations surgical volunteer organizations (≤50% donations of previous year; *n* = 17) and high donations surgical volunteer organizations (≥75%; *n* = 24). Univariate analyses were used to compare the two cohorts.

**Results:**

Of responding surgical volunteer organizations, 34 (83%) were unable to maintain full functionality due to COVID-19; 27% of high donations vs. 0% of low donations surgical volunteer organizations (*p* = 0.02). The three leading obstacles were finances/donations (78%), fewer volunteers (38%), and inadequate personal protective equipment (30%). In response, 39% of surgical volunteer organizations developed novel E-volunteering opportunities. For support, 85% of surgical volunteer organizations suggested monetary donations, 78% promotion through social media platforms, and 54% donation of personal protective equipment.

**Conclusion:**

The majority of surgical volunteer organizations were unable to maintain full functionality due to stressors caused by COVID-19, including limitations on finances, volunteers, and personal protective equipment.

## Introduction

The ongoing coronavirus disease (COVID-19) pandemic has presented unforeseen obstacles for nearly every industry but has arguably impacted none more so than the healthcare industry, both domestically and abroad. The WHO identified a shortage of 4.3 million healthcare workers globally in 2011, which has been exacerbated by the pandemic and expected to increase to 18 million by 2030 (1, 2). This personnel deficiency disproportionately affects developing countries, as doctor-patient ratios in most African countries are ~1:10,000 compared to those of developed countries such as the United States (1:277), Italy (1:270), and Germany (1:417) (3).

Low- and middle-income countries (LMICs) rely largely on the efforts of international volunteers and organizations to meet their increasing healthcare demands. Over the years, surgical volunteer organizations (SVOs) have collaborated with host countries to overcome hurdles and improve the quality and availability of care in low-resource settings (4, 5). High-income countries are able provide assistance to LMICs through donations, teaching and training of local providers, disaster relief, assistance with capacity-building and task-shifting, and provision of surgical services to an otherwise neglected, underserved community (6). In 2016, 200,000 American global health volunteers traveled around the world to provide medical assistance with an associated annual expenditure of $250 million (7, 8). However, the COVID-19 pandemic has forced many SVOs to shut down temporarily or permanently (9). This has heightened implications for LMICs whose surgical volume is largely urgent and non-elective (9). To our knowledge, no previous studies have sought to understand the impact of COVID-19 on SVOs.

The purpose of this study was to survey international SVOs to identify obstacles the ongoing pandemic has presented and to elucidate solutions that have been implemented to maintain functionality.

## Materials and Methods

The present study was a prospective survey study of SVOs with at least one branch of operation in the United States or Canada. Organizations meeting this criteria were identified through previously published lists (10, 11) as well as further inquiry through online and social media searches querying for relevant keywords, yielding 156 potential participants. A 15-question, web-based survey (Appendix) was devised by physicians with extensive international surgical volunteering experience and distributed *via* SurveyMonkey (SurveyMonkey Inc., San Mateo, California, USA). The survey was active from November 2020 to January 2021 and was re-sent to initial non-respondents.

The survey included questions about fundraising and maintaining volunteer operations as well as the use of e-volunteering, telecommunications, and social media. Questions were asked in relation to the start of the COVID-19 pandemic, which was defined as March 11, 2020 per the WHO (12). Responding SVOs were separated into two groups based on the percentage of donations received in 2020 vs. 2019: low donations SVOs (≤50% of their previous year's annual donations) and high donations SVOs (≥75% of their previous year's annual donations).

Descriptive statistics were generated. Chi-squared tests were utilized to compare proportions as appropriate. Given that only 156 potential participants were identified and some of these organizations were expected to be nonoperational at time of the survey, a small sample size was considered acceptable for simple statistical methodology. All analyses and plot creation were conducted using SAS 9.4 (SAS Institute, Cary, NC). Alpha was set at 0.05.

## Results

Of the 156 SVOs identified, 41 completed the survey (26%). There was a 100% completion rate for those who accessed the survey link. Thirty-four (83%) of responding SVOs were unable to maintain the full functionality of their organization in 2020 as a result of COVID-19.

### COVID-19's Impact on SVO Donations

**Figure 1:**
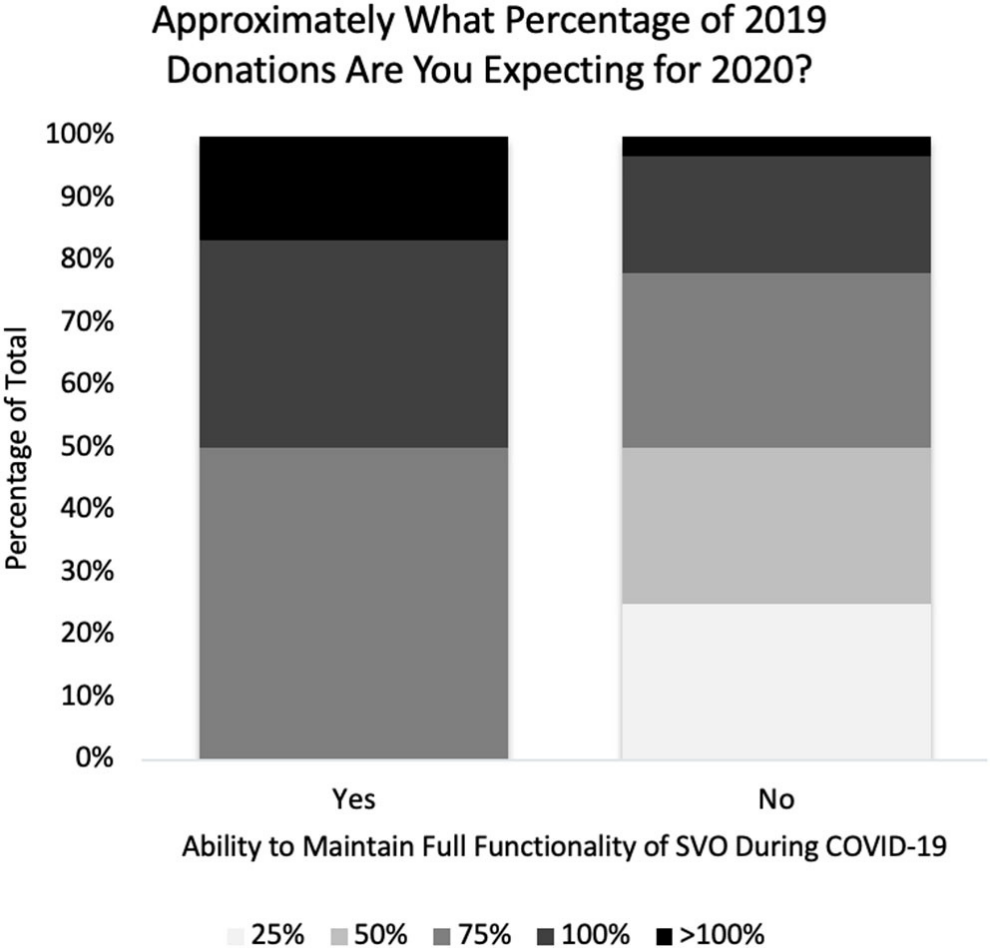
Change in annual donations vs. ability to maintain surgical volunteer organization functionality during the pandemic.

In 2020, 21% of SVOs received 25% of their previous year's donations, 21% received 50%, 32% received 75%, 21% received 100%, and 5.3% were able to acquire >100% of their previous year's donations. Seventeen were considered low donations SVOs and 24 high donations SVOs. Twenty-seven percent of high donations SVOs were able to maintain their organization's full functionality in 2020 vs. 0% of low donations SVOs (*p* = 0.02) (Figure 1).

**Figure 2:**
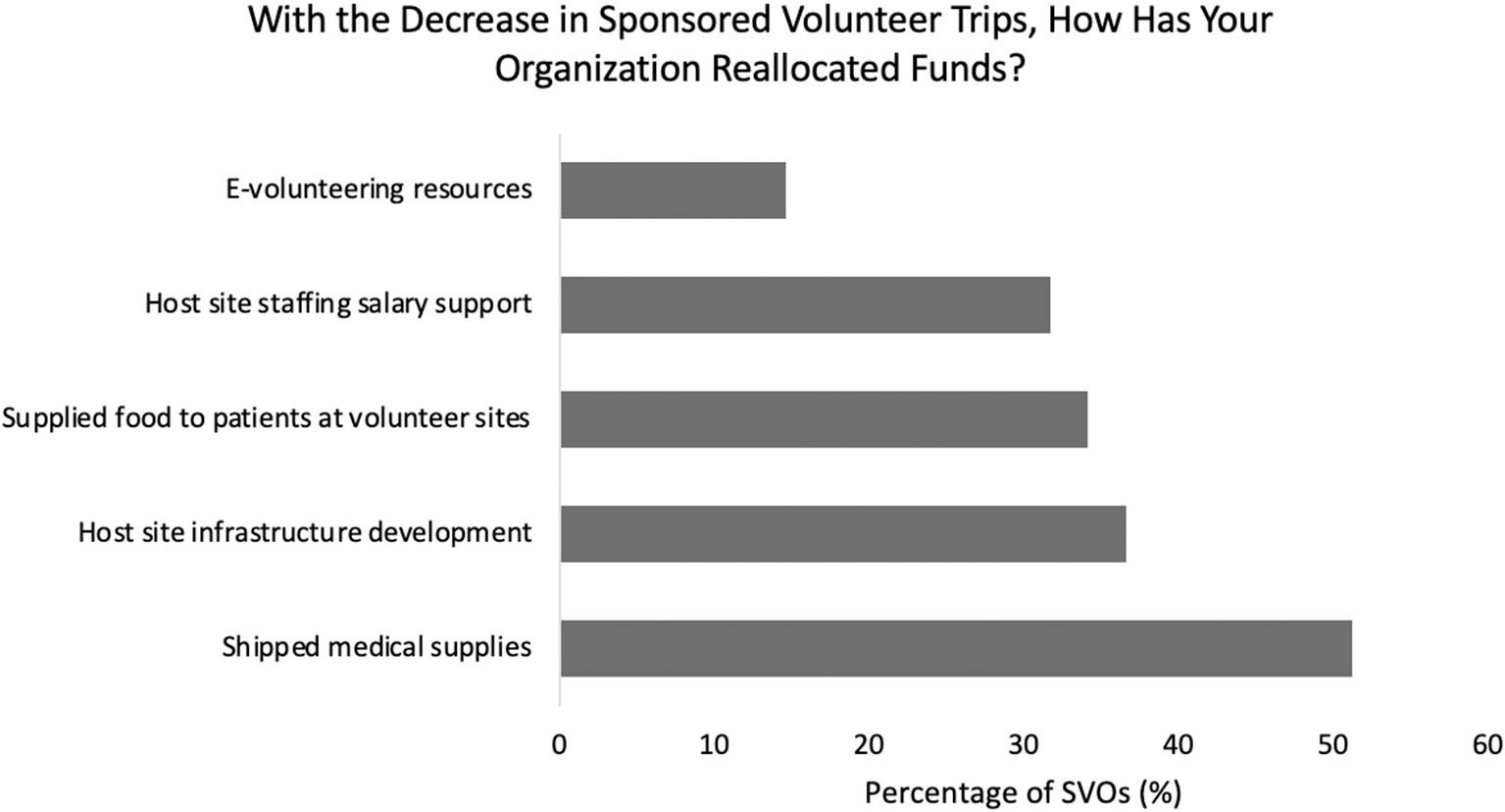
Breakdown of funding reallocation by surgical volunteer organizations during the pandemic.

With nearly a full cessation of sponsored, international volunteer trips during the pandemic, SVOs redistributed funding toward shipping of medical supplies (53%), provisions available for patients at volunteer sites (35%), host site staffing salary support (33%), host site infrastructure development (38%), and e-volunteering resources (15%) (Figure 2). Amongst the high donation SVOs, 59% reallocated funds to host site infrastructure development, whereas only 13% of low donation SVOs invested similarly (*p* < 0.01). Fifty-five percent of SVOs placed a temporary pause on all their organization's volunteer efforts.

**Figure 3:**
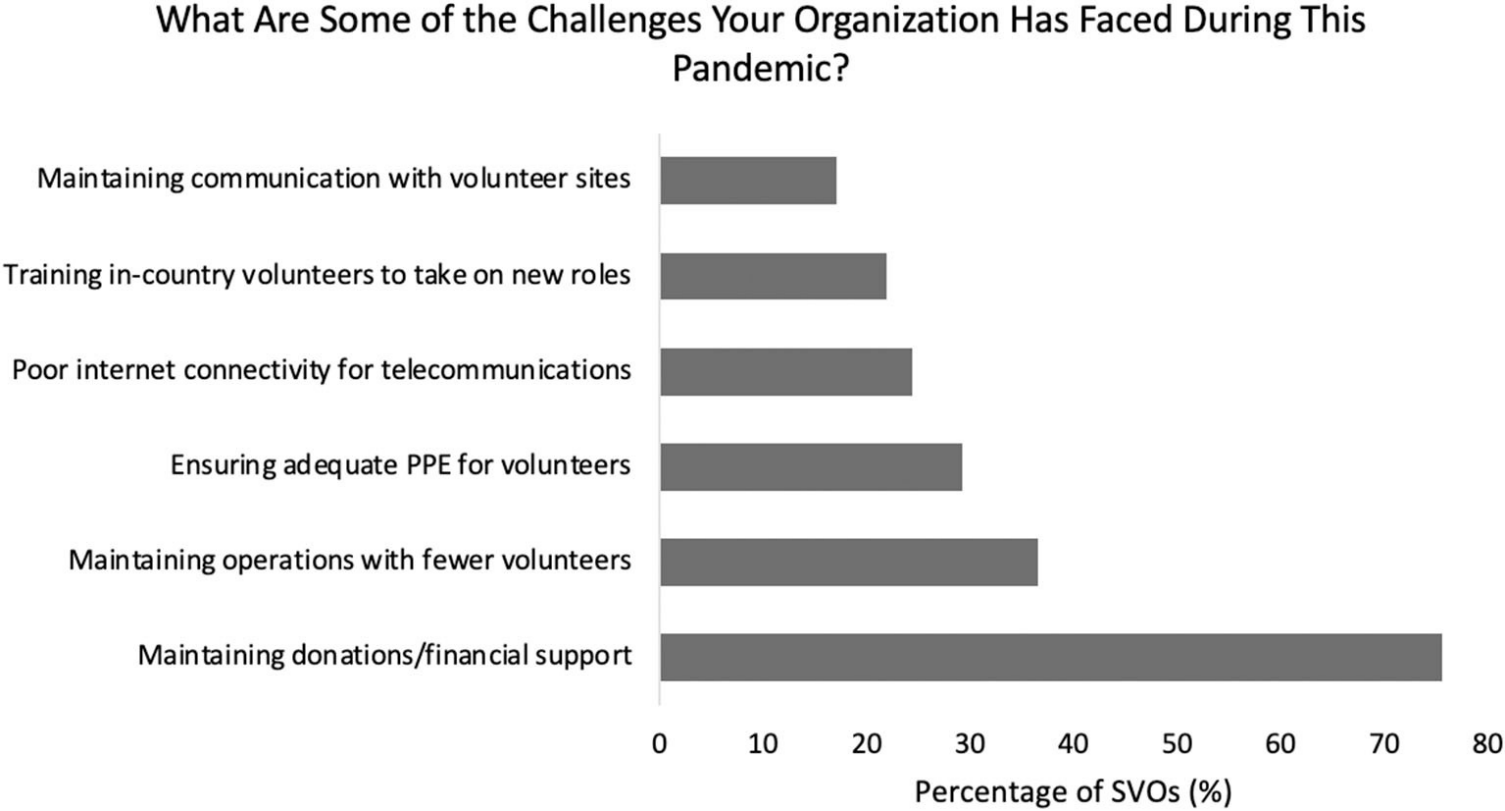
Breakdown of challenges faced by surgical volunteer organizations during the pandemic.

Regarding challenges encountered during the pandemic, SVOs struggled to maintain donations and financial support (78%), continue operations with fewer volunteers (38%), ensure all volunteers had adequate personal protective equipment (PPE, 30%), sustain internet connectivity to support telecommunications (25%), train in-country volunteers to take on new roles (23%), and preserve ongoing communications with volunteer sites (18%) (Figure 3). Forty-six percent of high donations SVOs described limitations with internet connectivity for telecommunications, while no low donation SVOs encountered this obstacle (*p* < 0.01). There was no significant difference between high donations (32%) and low donations SVOs (33%) in perceived need to develop host site infrastructure supporting telecommunications (*p* = 0.92).

### COVID-19's Impact on SVO Personnel

**Figure 4:**
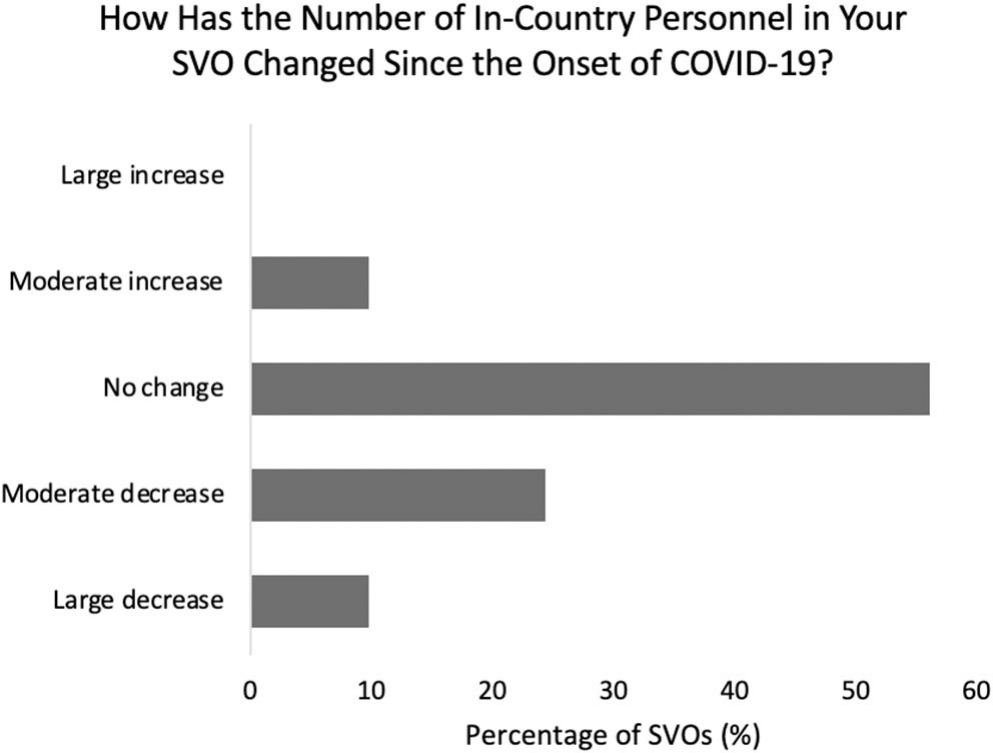
Changes in surgical volunteer organization personnel during the pandemic.

Thirty-four percent of survey respondents indicated a moderate to large decrease in their organization's total number of “in-country” personnel since the onset of COVID-19. Fifty-six percent noted no change, and 9.8% stated a moderate increase in personnel (Figure 4). Organizations that experienced a greater decrease in local personnel faced greater challenges maintaining operations (*p* = 0.02). Twenty-five percent of SVOs with a moderate increase and 22% with no changes in personnel numbers indicated struggles maintaining operations. While 50% of SVOs with large personnel reductions and 67% with moderate reductions felt a need to development telecommunications infrastructure, 17% with no changes and 0% with a moderate increase in personnel indicated similar responses (*p* = 0.02).

### Recommendations and Innovations by SVOs

**Figure 5:**
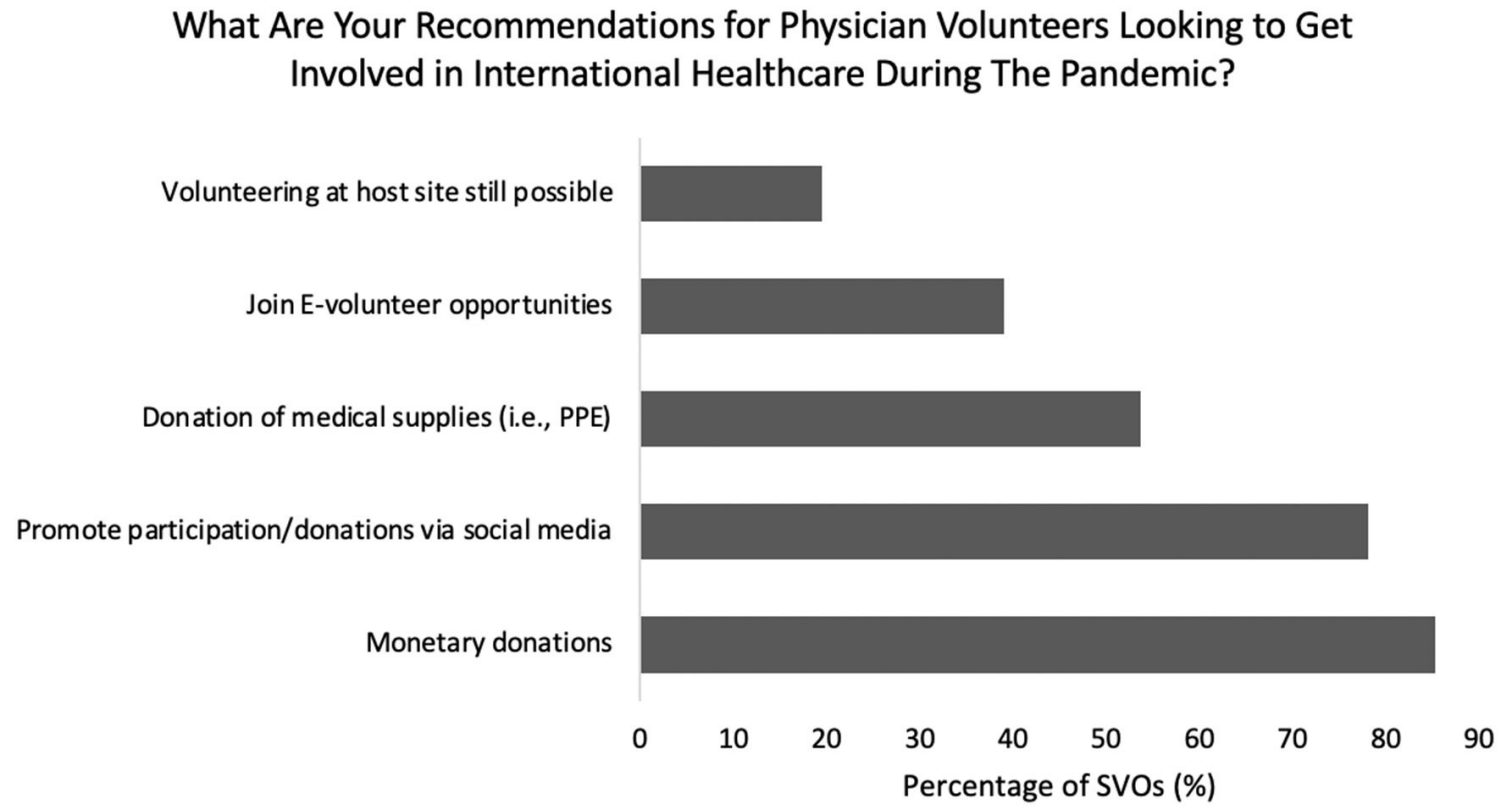
Breakdown of recommendations for physician volunteers during the pandemic.

**Figure 6:**
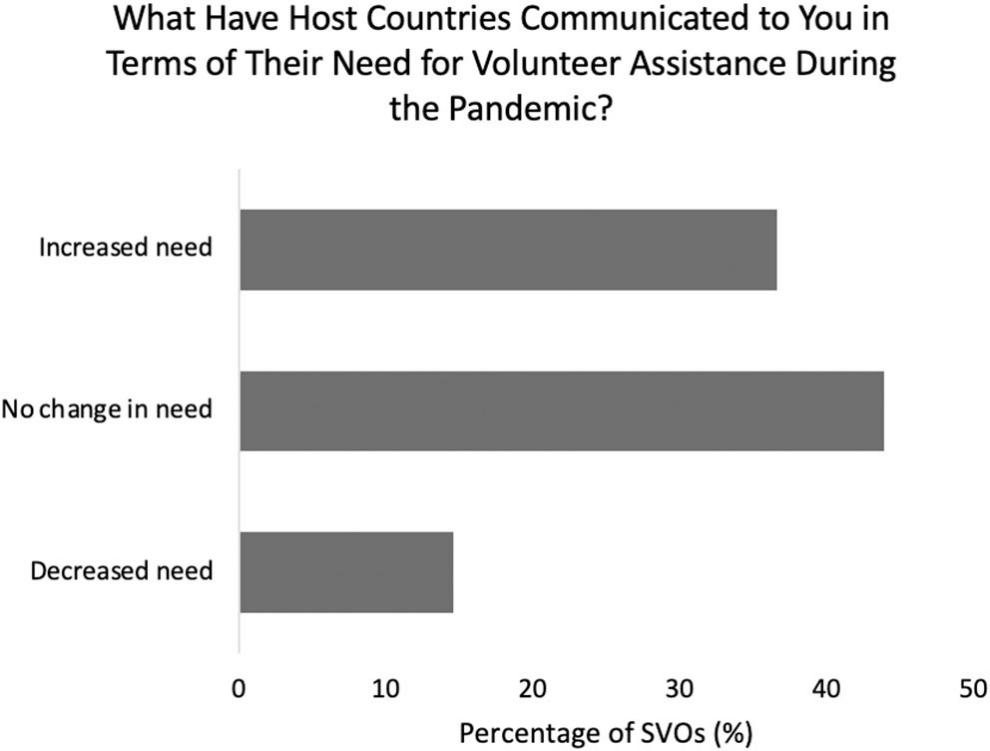
Changes in host country need for volunteer assistance during the pandemic.

Responding SVOs indicated the following as ways physician volunteers can contribute during the pandemic: monetary donations (85%), promoting participation and donations through social media platforms (78%), donation of medical supplies (e.g., PPE, 54%), and joining e-volunteering opportunities (39%) (Figure 5). Twenty percent noted that volunteering at the host site was still a possibility. Thirty-nine percent of partnering host countries communicated an increased need for surgical volunteers since the onset of the pandemic, 46% no change, and 15% a decreased need (Figure 6).

**Figure 7:**
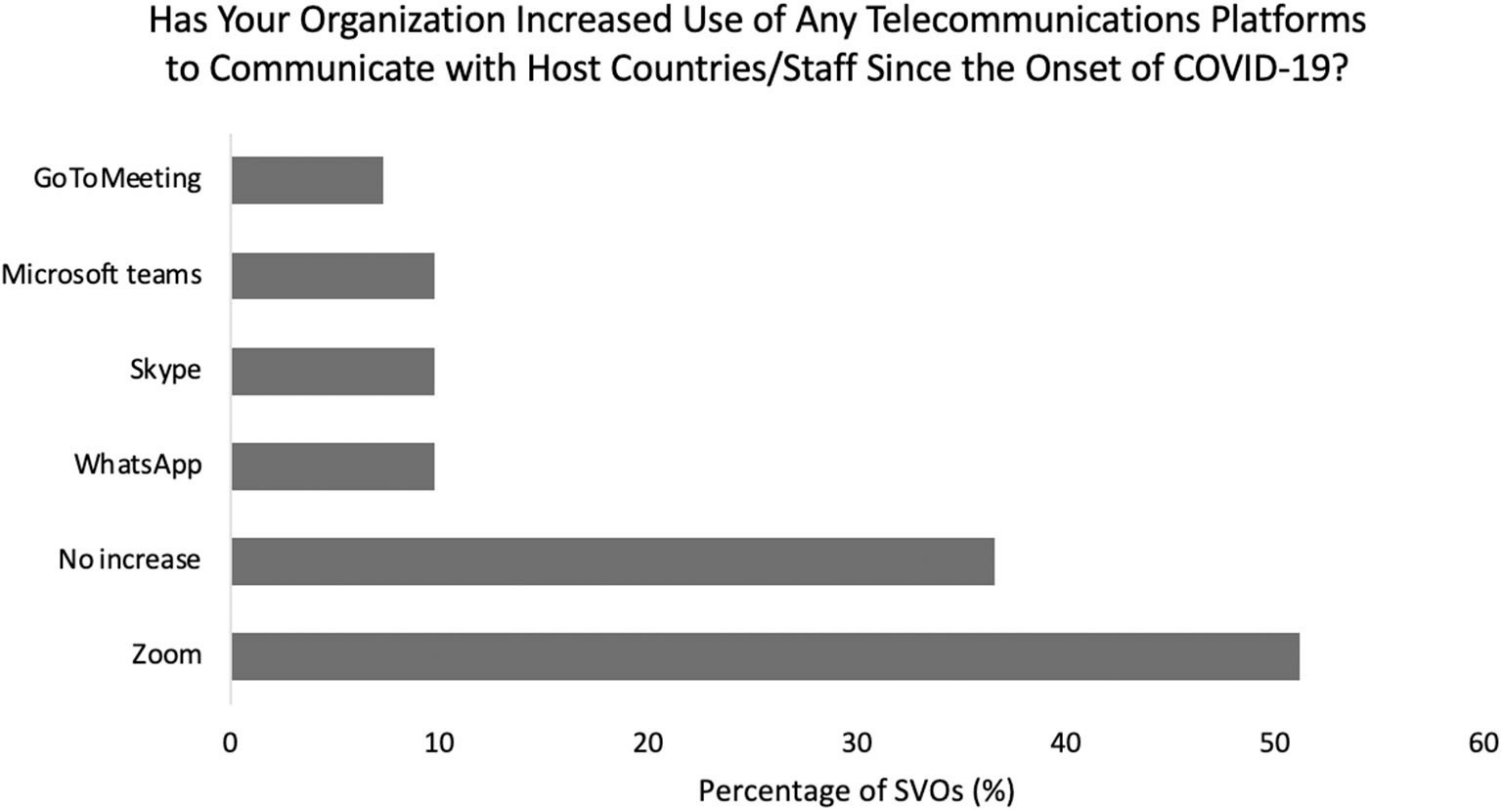
Breakdown of telecommunication platform utilization by surgical volunteer organizations during the pandemic.

**Figure 8:**
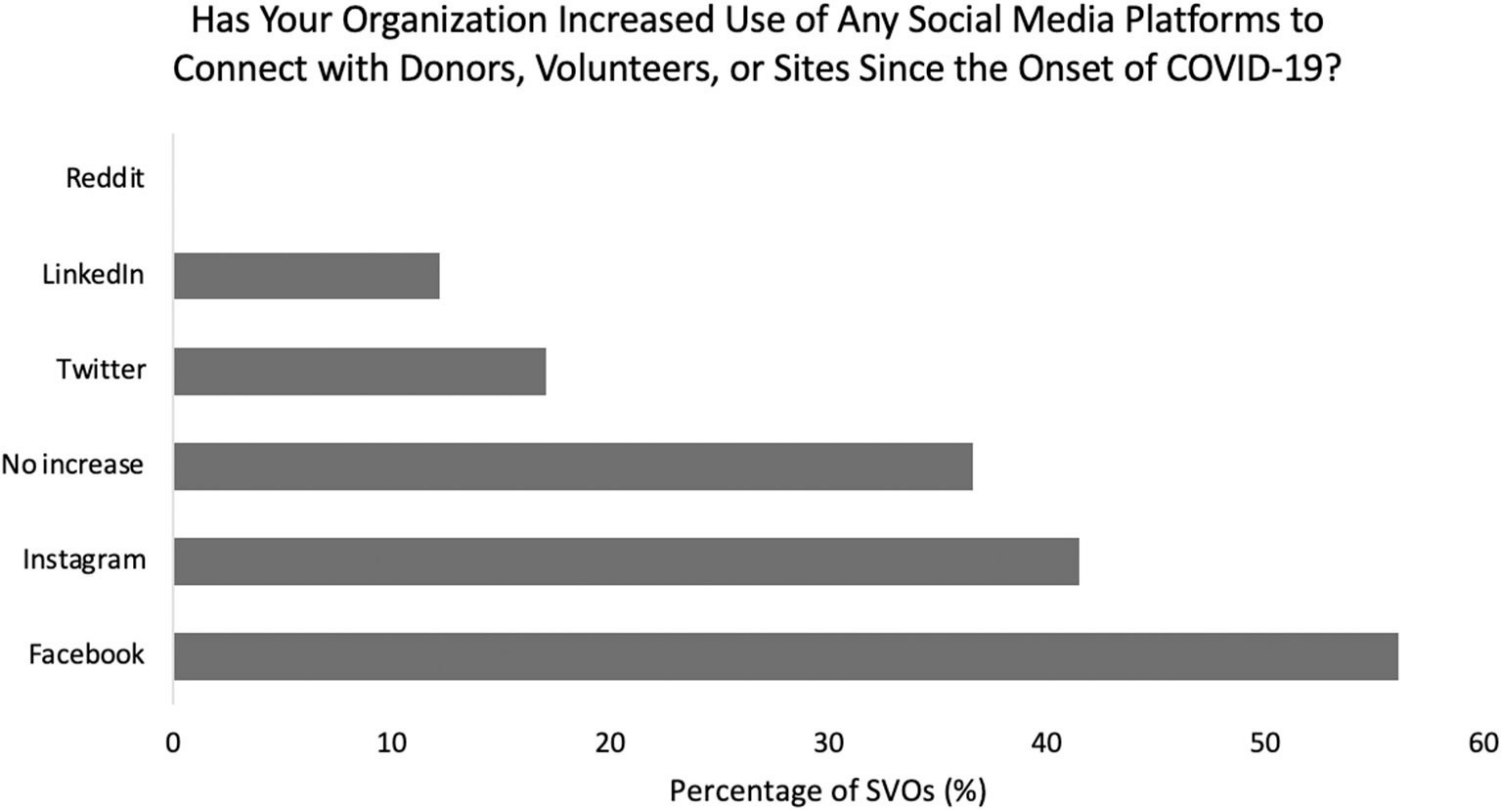
Breakdown of social media platform utilization by surgical volunteer organizations during the pandemic.

In response to COVID-19, 39% of SVOs developed e-volunteering opportunities. Amongst these organizations, the number of active e-volunteers ranged from 3 to 100 per SVO at the time of survey. Sixty-three percent of responding organizations increased their use of telecommunications with their host sites, most commonly Zoom (53%), WhatsApp (10%), Skype (10%), Microsoft Teams (10%), and GoToMeeting (7.5%) (Figures 5, 7). Additionally, 63% of SVOs increased their use of social media to connect with donors, volunteers, and volunteer sites since the start of the pandemic. The most common platforms were Facebook (58%), Instagram (43%), Twitter (18%), and LinkedIn (13%) (Figure 8).

## Discussion

The COVID-19 pandemic has presented unforeseen challenges for surgical volunteer organizations that have required adaptability to maximize functionality. With the majority of partnering host sites communicating similar or higher need of international assistance compared with pre-COVID levels, how can we continue to effectively contribute despite the constraints of the pandemic? To our knowledge, this study is the first to investigate the impact of the pandemic on international volunteering.

As suspected, an overwhelming majority of responding SVOs reported a diminished level of functionality in 2020 along with a substantial drop in their annual donations. Kobayashi et al. (13) proposed that a decline in international healthcare donations is expected when individuals have concerns about their home country's economy, as was prevalent throughout the United States during the pandemic. Our results highlight the importance of monetary donations for the functionality of international volunteer organizations. While over a quarter of SVOs that received ≥75% of their annual donations maintained full functionality during 2020, no SVOs that received ≤ 50% of their usual annual donations were able to. The three leading obstacles SVOs reported facing were finances and donations, operating with fewer volunteers, and ensuring adequate PPE for volunteers. This was consistent with predictions by Bong et al. (9) who posited that the negative effects of COVID-19 were expected to be more severe in LMICs due to deficiencies in medical providers, PPE, and medical supplies.

Even though many organizations were unable to maintain full functionality, SVOs discovered new and creative ways to stay involved with their host countries. Many organizations provided more humanitarian aid, including supplying shelters, blankets, clothes, food, hygiene products and other necessities. Others aimed to provide PPE to not only their healthcare workers but also patients and staff in neighboring hospitals. Despite varying nonoperational periods, 59% of high donations SVOs and 13% of low donations SVOs were able to reallocate funds to development of host site infrastructure in order to help partner countries better cope with the restraints of the pandemic.

Further, the majority of SVOs experienced either no change or an increase in the number of in-country personnel. With donations dropping at least 25% for most SVOs, it is unclear how organizations were able to retain and pay the salaries of host country staff. One SVO that managed to continue full operation ascribed their success to a decades-long history of missionary work and a deep local network with which they maintained open communications. Multiple other organizations stated that their LMIC volunteer activity is only augmented by, but not dependent on, foreign short-term volunteers. In fact, some note an increase in surgical volumes during the pandemic driven largely by “in-country” personnel. The efficacy of international SVOs that rely on short-term volunteers is frequently called into question, with previous studies citing the paucity of long-term outcomes and the potential creation of reverse innovation, or perpetuated dependency on high-income country volunteers (14, 15). Therefore, local capacity-building at host sites and an emphasis on independence has allowed sustainable operation during unprecedented times. It is also important to note that one SVO mentioned placing a greater emphasis on task-shifting, or the delegation of healthcare tasks to less specialized groups of local healthcare workers to increase access to care. This strategy has had expanding prevalence and success in LMICs and should be further explored by SVOs seeking to maximize their international productivity, as long as it can be implemented safely (2, 16, 17) ^1^.

In addition, 39% of responding SVOs developed new e-volunteering opportunities since the start of the pandemic and recommended them for physician volunteers hoping to get involved in international healthcare. E-volunteering is defined as “providing remote mentoring, research support, curricula development, case consultation, etc. through email, Skype, and other web-based technologies.” ^1^ The increased utilization of e-volunteering suggests a shift toward e-volunteerism in international medicine, as in concurrence with Tierney and Boodoosingh (18), and that remote assistance to LMICs may become more common post-pandemic. For example, Talsania et al. (7) noted that since the start of the pandemic, visiting volunteers in one orthopedic nonprofit organization have already developed new online teaching tools to benefit LMIC providers. However, expansion of such initiatives also brings additional challenges for remote locations with unreliable internet and technological access (18).

Moreover, as the general public increased reliance on telecommunications during 2020, so did SVOs (19). Interestingly, we found that high donations SVOs were more likely to both remain functional and face challenges with internet connectivity, indicating their usage of telecommunications, whereas no low donations SVO endorsed this limitation. A study by Mars (20) suggested that tele-education geared toward less experienced providers has much promise for LMICs in Africa, though such infrastructure is often the limiting factor in low resource settings. Thus, it is reasonable for resources to be diverted to the enhancement of telecommunications infrastructure, especially when volunteer trips are on hold. SVOs demonstrated an understanding of this, as there was a significant correlation between decreasing personnel at volunteer sites and perceived need for infrastructure development to support telecommunications. Overall, these findings demonstrate an increased reliance on telecommunications that will likely persist into the future.

In line with an increased role for telecommunications during COVID-19, many SVOs and their international partners turned to online platforms and social media as a means of training volunteers, connecting with donors and volunteers, continuing to provide medical oversight remotely, and networking (7). SVOs reported hosting virtual webinars, symposiums, weekly Zoom lectures, and education series with targeted training in surgical technique, patient and nursing care, and anesthesia. One organization described successfully increasing internet capacity at an African site to allow for more video calls with local management, while another SVO provided increasing remote radiology reading assistance and related teaching. Along a similar vein, social media is becoming an increasingly powerful and utilized platform for surgical volunteer organizations. Over 60% of responding SVOs had increased the use of social media platforms to connect with donors, volunteers, and volunteer sites since the start of COVID-19. Nearly 80% recommended promoting organizational participation and donations through social media platforms, Facebook and Instagram being the most popular options. Di Lauro et al. (21) reported that when social media is utilized correctly, organizations can increase transparency, display involvement and engagement, as well as improve organizational image.

There are several limitations to this study. First, the authors' choice to use only surgical volunteer organizations in the United States and Canada may lack international generalizability, but this minimizes the variability that may arise with unique country-to-country COVID-19 protocols. Second, SVOs were identified for inclusion in our analysis *via* a list generated by Ng-Kamstra et al. in 2015 (11), which used a web-based query of multiple databases to create a comprehensive listing of surgical, non-governmental organizations. Considering the five-year interval since the study's publication, the exhaustiveness of this list is uncertain. This strategy also prevented us from ascertaining the accuracy of listed contact information for these SVOs, making it difficult to quantify what proportion of our emailed surveys were accessible by the intended recipients. Nevertheless, the findings presented in the present study are likely to persist if additional SVOs were included given the universal nature of the COVID-19 experience. Finally, the low response rate invariably affected the power of our analyses, a known limitation of web-based surveys (22). However, the survey was completed by all SVOs that opened the email requesting participation with the majority of questions completed by the entire cohort, minimizing the interference of responder bias.

## Conclusion

The COVID-19 pandemic has been an unexpected stressor on the healthcare community, and the present study is one of the first to evaluate how international surgical volunteering has been affected. SVOs that placed an emphasis on host site capacity-building and relied less on short-term volunteers tended to maintain higher levels of functionality. Yet, the majority were unable to maintain full functionality. The large decrease in SVO functionality produces a magnitude of impact that is unquantifiable at this time but must be a source of future investigation. A better understanding of the obstacles, limitations, and innovative solutions for SVOs is imperative to mitigate the loss of established forward momentum in LMICs. Reduction in resources and personnel at hospitals during the pandemic may have fostered a reliance on less qualified providers (i.e., spiritual healers), which may result in global patients reacquiring distrust in the overburdened healthcare centers and practices of Westernized medicine. SVOs must thus strategize to overcome the obstacles of the pandemic to continue providing surgical services, educating and training local workers and perhaps most importantly, improve capacity-building in LMICs.

## Data Availability Statement

The raw data supporting the conclusions of this article will be made available by the authors, without undue reservation.

## Author Contributions

WD performed the data collection, statistical analysis, and wrote the initial manuscript. AX, WD, and SP contributed to data collection and contributed key sections of the manuscript. JO and JB contributed to the conception and design of the study and made critical revisions. All authors contributed to manuscript revision, read, and approved the submitted version.

## Conflict of Interest

The authors declare that the research was conducted in the absence of any commercial or financial relationships that could be construed as a potential conflict of interest.

## Publisher's Note

All claims expressed in this article are solely those of the authors and do not necessarily represent those of their affiliated organizations, or those of the publisher, the editors and the reviewers. Any product that may be evaluated in this article, or claim that may be made by its manufacturer, is not guaranteed or endorsed by the publisher.

## Footnotes

^1^ https://hvousa.org/get-involved/e-volunteering/
