# Supplementary material for: The Impact of the COVID-19 Pandemic on the Functionality of International Surgical Volunteer Organizations
Source: Front Surg. 2022 Apr 6;9:868023. doi: 10.3389/fsurg.2022.868023 (PMC9019131; doi:10.3389/fsurg.2022.868023)
Supplement: Supplementary file 1 [file Data_Sheet_1.pdf]

Supplementary Material

APPENDIX

Survey for Surgical Volunteer Organizations (Answer options listed directly below questions)

Fundraising and Maintaining Volunteer Operations

1. What is the name of your organization?
(Open response)

2. Have you been able to maintain the full functionality of your SVO during COVID-19?
a) Yes, b) No

3. Approximately what percentage of your 2019 annual donations are you expecting for 2020?
a) 25%, b) 50%, b) 75%, c) 100%, d) > 100%

4. With the decrease in sponsored volunteer trips, how has your organization reallocated funds?
(Mark all that apply)

a) Shipped medical supplies, b) Supplied food to patients at volunteer sites, c) Host site staffing salary support, d) Host site infrastructure development, e) E-volunteering resources, f) Temporary pause of volunteer organization efforts

5. What are some of the challenges your organization has faced during this pandemic?
(Mark all that apply)

a) Poor internet connectivity with telecommunications, b) Maintaining ongoing communication with volunteer sites, c) Maintaining donations and financial support, d) Ensuring adequate PPE for volunteers, e) Maintaining operations with fewer volunteers, f) Training in-country volunteers to take on new roles

6. What creative methods has your organization devised to stay involved in your host countries?
(Open response)

7. What are your recommendations for physician volunteers looking to get involved in international healthcare during this time?
(Mark all that apply)

a) Join e-volunteering opportunities, b) Volunteering internationally at host site is still possible (must do two-week quarantine after arrival), c) Monetary donations, d) Donations of medical supplies (i.e. PPE), e) Promote participation and donations through social media platforms, f) Other (please specify)

8. How has the number of "in-country" personnel in your NGO changed since the onset of COVID-19?
a) Large decrease, b) Moderate decrease, c) No change, d) Moderate increase, e) Large increase

9. How have the roles of international volunteers in your SVO changed since the start of the pandemic?
(Open response)

10. What have host countries communicated to you in terms of their need for volunteer assistance during the pandemic?
a) Decreased need for surgical volunteers, b) Same amount of need for surgical volunteers, c) Increased need for surgical volunteers

E-volunteering, Telecommunications and Social Media

11. Since the onset of COVID-19, has your organization developed any e-volunteering opportunities?
a) Yes, b) No

If yes, what new opportunities exist?
(Open response)

12. Approximately how many current e-volunteers or remote volunteers are participating within your organization?
(Open response)

13. Was there a need for infrastructure development after the start of COVID-19 at your volunteer sites to support the additional use of telecommunications?
a) Yes, b) No

If yes, were you able to meet those needs?
a) Yes, b) No

14. Has your communication with host countries and staff evolved to use more telecommunications software than before?
a) Zoom, b) Skype, c) Microsoft Teams, d) GoToMeeting, e) No increased use of telecommunications software, f) Other (open response)

15. Has your organization increased the use of any of the following social media platforms to connect with donors, volunteers and volunteer sites since the start of COVID-19?
a) Twitter, b) Instagram, c) Facebook, d) Reddit, e) LinkedIn, f) No increased use of social media outreach, f) Other (open response)
